# Police-reported family violence: are there differences amongst South Asian Australians and Australian-born Australians?

**DOI:** 10.1080/13218719.2023.2280517

**Published:** 2024-01-11

**Authors:** Heshani S. De Silva, Stephane M. Shepherd, Troy E. McEwan

**Affiliations:** a Centre for Forensic Behavioural Science, Swinburne University of Technology, Melbourne, VIC, Australia; b Department of General Practice & Department of Social Work, The University of Melbourne, Melbourne, VIC, Australia; c Alfred Deakin Institute for Citizenship and Globalisation, Deakin University, Burwood, VIC, Australia

**Keywords:** Australia, family violence, migrants, partner violence, South Asian

## Abstract

Numerous qualitative studies report South Asian migrants use police as a last resort for family violence (FV), however no quantitative evidence exists in Australia. This study examines police-reported FV recorded by Victorian police between September 2019 and February 2020 (*N* = 32,450) and compares reports made by South Asian-born (SAB) Australians to Australian-born (AB) Australians. Demographics, incidence and revictimisation rates, severity, frequency of risk and vulnerability factors (e.g. isolation & visa dependency) reported by the two groups were examined. More females were affected family members (AFMs) for both groups; however, SAB males were more likely to report non-partner FV. SAB AFMs reported a lower incidence rate and occurrence of revictimisation than AB AFMs. There were no significant differences in the level of severity (based on charges at time of incident); however, significantly higher number of risk factors were present for SABs reporting intimate partner violence. Mixed results emerged in the frequency of factors.

Family violence (FV) occurs in every society and culture (Garcia-Moreno et al., 2006) but may present differently amongst different cultural groups (Archer, 2006; Couture-Carron, 2017; Satyen et al., 2019). Global migration patterns make FV a worldwide issue, thus the needs of different populations must be identified to allow for appropriate prevention and intervention responses. Migrants can face additional vulnerabilities compared to non-migrants (Ahmad et al., 2004; Midlarsky et al., 2006; Ozturk et al., 2019; Sripada, 2021). South Asians (defined as individuals born in India, Sri Lanka, Pakistan, Bangladesh, Nepal, Maldives and Bhutan) have the largest number of emigrants worldwide, with India alone being the largest contributor to international immigration (Migration Data Portal, 2021).

Reddy (2019) reviewed the current literature and estimated the prevalence of FV amongst the South Asian diasporic community as around 38–70.5%, compared to the global average of 33% (World Health Organisation, WHO, 2021). Despite these high estimates, South Asian migrants are less likely to use FV services than the host population (Colucci et al., 2021; Hyman et al., 2009). Several authors have observed that when South Asian migrants do seek formal support, the service responses often neglect the nuances of their experiences, including the numerous barriers to help-seeking that South Asian migrants face (Gill & Harrison, 2016; Harrison & Gill, 2018; Mahapatra & Rai, 2019; Sultana et al., 2023). Due to these barriers, South Asian migrants often contact the police as their last resort, when the violence has escalated and has become unbearably severe (Bhandari, 2018; Ghafournia & Easteal, 2021). As a result, it is possible that police reports made by South Asians could be more severe and present with greater immediate risk than cases involving people of other backgrounds. The aim of this study is, therefore, to describe police reports of FV by the Australian South Asian diaspora and contrast them to those of people born in Australia. Determining whether differences exist between these two groups may show whether South Asian Australians present with higher levels of severity and need.

### Family violence amongst South Asians

FV amongst South Asian migrants has been explored by researchers in Canada, the United States and the United Kingdom for more than 20 years. Although quantitative research on FV amongst South Asian migrants is limited due to non-representative samples, their findings suggest that South Asian migrants are disproportionately impacted by FV due to additional challenges they face (Reddy, 2019; Sabri et al., 2018). There is no equivalent Australian research, despite South Asians being one of the fastest growing migrant populations in Australia (Australian Bureau of Statistics, ABS, 2022a, 2022c). The limited nature of Australia’s research may have hindered the overall identification of South Asian Australian migrants as a vulnerable population.

The immigrant experience comes with a range of additional challenges that increase susceptibility to FV. For example, migration has been associated with increased risk of isolation and visa difficulties, both factors observed in samples of South Asian migrants experiencing FV (Bhandari, 2018; Menjivar & Salcido, 2002; Segrave, 2017). Isolation can be both social and physical (Tillyer & Wright, 2014), with migration involving physical distancing from original support networks as well as obstacles in forming new connections (e.g. due to a foreign/new language and culture). In a sample of South Asian women who had emigrated to the United States, increased social isolation was associated with increased severity of intimate partner violence (IPV; Raj & Silverman, 2002). Isolation can also increase an individual’s reliance on their partner, on whom they may also be dependent for residency status (Abraham, 2000; Dasgupta, 2000; Kallivayalil, 2010; Menjivar & Salcido, 2002). Qualitative research with South Asian migrant women in Australia suggests that visa dependency has been used as a tool to threaten and control them, as well as to influence their confidence in seeking help, including from police (Midlarsky et al., 2006; Segrave, 2017).

The patriarchal belief system of South Asian cultures contributes to the vulnerability of South Asian migrants by heightening their likelihood of the ‘severe manifestation of patriarchy’ (Ahmad et al., 2004, p. 264) – honour-based violence and dowry abuse. Honour-based violence is thought to be an extreme form of policing and controlling behaviour to avoid transgression against role expectations – for example, gender, cultural, societal or religious (Gill, 2014). While both men and women can be targets of honour-based violence, the majority of victims are women, as they are traditionally responsible for the household’s reputation through conformity to social norms and traditions (Gill, 2014; Lima et al., 2020). On the other hand, dowry, being the transfer of assets from the bride’s family to the groom’s, can lead to violence by either punishing a new bride for inadequate dowry or pressuring them for more dowry (Gangoli & Rew, 2011; Kumari, 1989; Martin et al., 2013). The dowry-abuse is often perpetrated by an intimate partner and/or by in-laws, whilst honour-based violence can be perpetrated by any family member (intimate partner, in-laws or biological family). Gradual recognition that these forms of violence exist within South Asian migrants has seen an emergence of FV cases being identified in Australia in recent years (Asher, 2020; Evlin, 2020a, 2020b, 2021; Singh, 2017, 2019). Unfortunately, both can escalate to fatal violence.

The household composition of many South Asian migrant families may heighten their risk of experiencing non-partner family violence (NPFV). That is, South Asian migrants are more likely to live in multi-generational households than non-migrants, which increases their level of exposure to potential perpetrators (Kanagaratnam et al., 2012; Mahapatra & Rai, 2019; O’Connor & Lee, 2022; Rai & Choi, 2022). The dynamics of a multi-generational household are complex; however, patriarchal values held by South Asians often dictate the household hierarchy (Martin et al., 2013). Once a South Asian woman is married, they may move in with the groom and his parents (Martin et al., 2013; Rastogi & Therly, 2006). Or equally, the eldest son of a South Asian family is required to look after his parents by living with them (Martin et al., 2013). Within these households, patriarchal gender roles of men controlling women are seen to persist in the broader family network, whereby fathers, brothers and male in-laws have control over a female relative (Hague et al., 2010; Martin et al., 2013). A hierarchy also exists between females, as daughters-in-law are sometimes abused by a mother-in-law, as the daughter-in-law often holds the lowest position in the overall hierarchy of a multi-generational household (Gangoli & Rew, 2011; Kandiyoti, 1988; Martin et al., 2013; Raj et al., 2006, 2011). Sometimes this control includes monitoring behaviours, with some South Asian migrants reporting that their partners and/or their in-laws have attempted to isolate them by preventing them from contacting friends and family overseas (Abraham, 2005; Ghafournia, 2011; Sabri & Young, 2022; Singh, 2019; Singh & Sidhu, 2020).

### Help-seeking amongst South Asian migrants

In addition to their heightened vulnerability, South Asian migrants have reported a range of barriers to help-seeking. One of the first barriers that South Asians face is limited recognition of the problem. For example, Finfgeld-Connett and Johnson (2013) found that South Asian women tend to react to experiences of FV with denial, minimalisation or acceptance. Yet when South Asian women and other migrants do recognise their experiences as FV, only a small proportion seek help, with a strong preference for informal support (Dehingia et al., 2022; Ozturk et al., 2019; Raj & Silverman, 2007; K. Tonsing & Tonsing, 2019; Yingling et al., 2015). For example, out of 44 South Asian migrants living in Boston who were victims/survivors of IPV, 53% reported having never accessed support or services, yet for those that did, the majority sought informal support (Raj & Silverman, 2007). Equally, in a sample of 49 South Asian migrants in Hong Kong, 75.5% reported seeking some sort of help – yet only one participant sought formal help (K. Tonsing & Tonsing, 2019).

Research has highlighted several barriers to formal help-seeking in this population. Part of the problem is a lack of culturally competent services. In a systematic review and meta-synthesis conducted by Sultana et al. (2023) on the barriers and facilitators of help-seeking for South Asian women in high-income countries, overall mainstream organisations were found to be limited by ‘lack of funding and lack of staff trained to understand and accommodate Asian needs and experiences’ (p. 3195). Similarly, in Australia, the 2016 Victorian Royal Commission on Family Violence indicated that culturally and linguistically diverse (CALD) populations are disproportionately impacted by FV and noted that their unique needs (e.g. need for interpreters or access to services regardless of visa status) are not being met by current service provision (State of Victoria, 2016). Other system-based barriers to formal help-seeking amongst South Asian migrants include distrust of formal services, uncertainty around outcomes such as unwanted outcomes (Belur, 2008; Hague et al., 2010; Merchant, 2000; Raj & Silverman, 2002, 2007), family disruption resulting from formal service interventions (Finfgeld-Connett & Johnson, 2013; Kanagaratnam et al., 2012; Mahapatra & Rai, 2019), threats to immigration status (Bhandari & Sabri, 2020; Femi-Ajao et al., 2020; Gill, 2004; Lodhia, 2010) and discriminatory practices (Australian Institute of Criminology, AIC, 2005; Belur, 2008; Ghafournia & Easteal, 2021; Gill, 2004; Izzidien, 2008; Liang et al., 2005; Rai & Choi, 2022; Raj & Silverman, 2002; Römkens, 2006; Sabri et al., 2018; Sultana et al., 2023; Yoshihama et al., 2011).

Qualitative studies demonstrate that when South Asian migrants do seek formal help, they do so because they perceive few other options. In a systematic review of coping strategies, Ozturk et al. (2019) indicated that South Asians tended to only use formal supports such as the police in specific situations: when they were ready to leave the perpetrator, when they were in a life-threatening situation and/or when they felt it became unbearable for themselves or their children (Ahmad et al., 2009; Kanagaratnam et al., 2012; Raj & Silverman, 2007). The literature suggests a relationship between help-seeking action and severity of FV, whereby those who had less severe injuries were less likely to seek help or they only sought help from informal sources – only if the abuse became more severe did they seek more formal sources (Barrett et al., 2020; Liang et al., 2005). Studies with South Asian populations have supported this suggestion, yet the majority of these studies were qualitative and used convenience samples (Bhandari & Sabri, 2020; Finfgeld-Connett & Johnson, 2013; Mahapatra & Rai, 2019; Raj & Silverman, 2007). If there is a tendency for South Asian migrants to avoid police help-seeking unless they deem their experience of violence as severe, police reports of FV amongst South Asian migrants should be less frequent but more severe in nature than reports by non-migrants. There has been no prior research that tests this hypothesis.

### Current study

At present, there is no quantitative research examining police-reported FV by South Asian migrants globally. The current study aimed to describe a population of FV reports made to Australian police by South Asian migrants, and to compare these to the population of reports made during the same period by people born in Australia. First, we described the demographic factors of those who reported FV to the police amongst South Asian-born (SAB) Australians and Australian-born (AB) Australians for overall FV, IPV and NPFV. Although the demographic examination was largely exploratory in nature, it was hypothesised that females would report more FV (including IPV and NPFV) across both populations.

Secondly, this study aimed to identify whether there were differences in the rate of reporting to police, as well as reported revictimisation. Due to the aforementioned barriers in help-seeking, it was hypothesised that South Asian Australians reporting to the police would demonstrate a lower incidence rate of reported incidents and lower rates of revictimisation. Finally, this study aimed to identify whether there are differences between SAB and AB individuals in the severity of FV reported. Due to qualitative reports indicating that South Asian migrants only report to the police as a last resort when the violence has become severe, it was hypothesised that South Asian migrants would demonstrate a higher level of severity in their police-reported FV incidents. Severity was operationalised in two ways, by comparing (a) the presence of a charge for significant physical violence or psychological abuse linked to the Family Violence Report (FVR) and (b) the number of risk and vulnerability factors recorded as present at the time of the police report.

This paper also aimed to examine whether SAB individuals were more likely than AB individuals to have specific risk factors associated in prior research with overall FV, or specifically with IPV or NPFV. For IPV it was hypothesised that SAB individuals would more frequently report being isolated, being visa dependent, being abused for longer than a month or experiencing escalating violence. They were also hypothesised to be more likely to have children present, to live with their respondent and be more likely to report risk factors associated with IPV femicide (Spencer & Stith, 2020). For NPFV, it was hypothesised that South Asian-born individuals would more frequently report being isolated, being financially strained, experiencing abuse for longer than a month, residing with their respondent, reporting jealous and controlling behaviours or experiencing escalating violence.

## Method

### Material

This study used 6 months of data on family reports made to police in the Australian state of Victoria (population of 6.5 million in 2020). FV was defined by the presence of an FVR recorded by Victoria Police. Victoria Police is the primary law enforcement agency that provides the full range of policing services and responses to the Victorian community, including all police FV responses. Any incident that involved FV was defined as such by the *Family Violence Protection Act 2008* (Vic) and is recorded as an FVR as a matter of police policy. The Act specifies FV as:

Behaviour by a person towards a family member of that person if that behaviour is physically or sexually abusive; or is emotionally or psychologically abusive; or is economically abusive; or is threatening; or is coercive; or in any other way controls or dominates the family member and causes that family member to fear for the safety and wellbeing of that family member or another person. (Family Violence Protection Act 2008, 2020, p. 16)

Between 1 September 2019 and 29 February 2020, there were 32,450 FVRs recorded by Victoria Police. For each FVR police have to identify a primary affected family member (AFM or victim) and a primary respondent (or perpetrator). They are required to collect information about both the AFM and the respondent as well as information about their relationship and prior FV guided by a structured risk assessment (more information about this risk assessment is available in Spivak et al. (2021). Table 1 summarises the risk factors coded by police at the time the FV is reported to them.

**Table 1.** Data extracted for each family violence report.

Category	Variable extracted from dataset
Demographic factors	Age of AFM
	Gender of AFM
	Country of birth of AFM
	Country of birth of respondent
	LGA of incident
Revictimisation	By same respondent
	By different respondent
AFM factors	Mental health problems
	Isolated
	Visa dependency
Respondent factors	Mental health problems
	Previous violent offence charge
	Previous physical violence towards anyone
	Unemployed
Relationship factors	Financial problems
	Presence of children
	Reside together
	Recently separated
	Abuse lasted for more than a month
	Recent escalation of violence
	History of family violence between AFM and respondent
Factors associated with severe outcomes	Jealous and controlling respondent
	Respondent ever threatened to harm or kill AFM
	Respondent physically violent whilst AFM pregnant
	Respondent ever or attempted to strangle/choke AFM
	Respondent ever sexual assaulted AFM
	Respondent ever stalked or harassed AFM

Note: AFM = affected family member; LGA = Local Government Area.

Risk factors for severe IPV were identified based on the results of Spencer and Stith’s (2020) meta-analysis of risk factors associated with intimate partner femicide. All risk factors identified as having odds ratios of greater than 2.0 in the meta-analysis were included in this study (Spencer & Stith, 2020).

The incidence rate analyses used the entire 6-month dataset of FVRs. The number of incidents were divided by Victoria’s estimated residential population and then multiplied by 100,000. Australia’s estimated residential population was provided through Census data, which is collected every 4 years (except for 2020 due to COVID-19). The 2021 Census reported a population of 6.5 million in Victoria.

Demographic, revictimisation and the severity analyses used the first incident reported by the AFM within the 6-month reporting period. The first incident will henceforth be called the ‘reference incident’.

### Design

The SAB sample was classified as any AFM who had their country of birth as any of the following six countries: India, Sri Lanka, Pakistan, Bangladesh, Nepal or Bhutan. The AB sample only consisted of AFMs born in Australia. Respondents were categorised into these same categories; however, those who were born elsewhere or did not have a country of birth (COB) reported were incorporated into an ‘Other-Born’ category.

The type of relationship reported was categorised by the relationship between the dyad involved, which was either ‘Intimate Partner Violence (IPV)’ (which included married, de facto, dating, separated/broken up and divorced relationships) or ‘Non-Partner FV (NPFV)’ (which included parent or parental relationships, siblings, other family relationships or kinship relationships). Analysis examining overall FV was conducted by a combination of both IPV and NPFV incidents.

Demographic data were coded into specific variables to facilitate the examination of the risk and vulnerability factors related to the different types of FV. First, each Local Government Area (LGA) captured by Victoria Police was matched and coded onto Levels 1–5 according to the Australian Bureau of Statistics (ABS) Index of Relative Socio-Economic Advantage and Disadvantage (IRSAD; ABS, 2018). The coded LGAs were then coded into the database on SPSS to produce a Socio-Economic Indexes for Areas (SEIFA) variable. Second, AFM age was coded into four categories, ‘Under 25’, ‘25–34’, ‘35–49’ and ‘50+’.

Revictimisation was present if an AFM had at least one additional FVR in the 12 months following their reference incident. The calculations for revictimisation by a different respondent were coded by taking the number of times an AFM reported an FVR with the same respondent in the 12 months following their reference incident and subtracting it from the number of times an AFM reported an FVR with any respondent within the 12 months.

FVRs were classified as ‘severe’ if on-site police officers indicated they intended to charge a respondent with one or more of the following: violent offences (indictable physical assault, homicide, armed robbery, robbery, aggravated burglary, false imprisonment/kidnap); stalking; threats to harm or kill; sex offences; arson causing death or endangering life; or driving offences causing death or endangering life. Even where charges were not authorised to proceed by a senior officer, they were included in ‘severe’ coding to provide the broadest possible identification of FV behaviour that was likely to cause significant harm.

### Statistical analysis

Data were analysed using IBM SPSS Statistics Version 28.0.1.1 (15). Chi-square analysis was used to compare SAB individuals and was compared to AB individuals by the type of FV reported and the sex of the AFMs reporting each type.

The incidence rate was calculated by dividing the total number of incidents reported by each sample population across the 6 months (SAB and AB) by their estimated residential population. This was then multiplied by 100,000. The estimated residential population was informed by the 2021 Census data, which stated that there were 387,763 SAB individuals and 4,228,684 AB individuals living in Victoria at the time (ABS, 2021, 2022b).

The dataset was then subset to only include reference incidents, allowing for analysis by AFM (i.e. by person) rather than by incident. The incidents where the two parties had a carer relationship (either as the AFM or the respondent) or co-resident relationship were excluded. Chi-square analyses, with Cramer’s *V* as a measure of effect size, were used to test for demographic differences between the AFMs from the two different populations, both for the overall sample population and for each sample within the two different types of FV (IPV and NPFV).

Three different 1:2 case-control matched samples were then created for SAB AFMs and AB AFMs based on sex, age and SEIFA: one for the overall sample, one for the sample of AFMs who reported their reference incident as an IPV incident and another for the AFMs who reported an NPFV incident. The case-control matches allowed for demographic factors to be controlled for in subsequent analyses.

Chi-square analyses with odds ratios (95% confidence interval, CI) as an effect size measure were used to identify whether there was a difference between the two groups in: (a) reporting revictimisation in the 12 months following the reference incident, (b) the proportion of FVRs associated with the presence of a charge for significant physical violence or psychological abuse and (c) the frequency of each risk and vulnerability factor. A Bonferroni correction was used to control error associated with a large number of comparisons occurring within each family of analyses.

A variable was created representing the number of risk and vulnerability factors recorded as present in each FVR: 20 risk factors were used to calculate this score for overall FV, 15 for IPV and 7 for NPFV. A Mann–Whitney *U* test was then used to compare the number of risk and vulnerability factors experienced between the two groups for each type of violence. The effect size was estimated using the equation proposed by Field (2009; Tomczak & Tomczak, 2014).

## Results

### Sample characteristics

Of 388 FVRs involving SAB, 380 (97.94%) involve unique AFMs. The AB population had 15,562 FVRs, of which 14,808 (96.15%) involved unique AFMs. There were no significant sex differences between the two populations of AFMs, χ^2^(1) = 1.685, *p* = .194. However, there were significant age differences between the two populations, χ^2^(3) = 77.22, *p* < .001, with AB individuals more evenly distributed across the different ages, whilst SAB individuals were more clustered around the 25–49-year age group. There were also significant differences in SEIFA levels, χ^2^(4) = 116.480, *p* < .001, with most SABs in the two top quintiles (73.5%), whilst ABs were spread more evenly from the second quintile to the most advantaged.

#### Sample characteristics of AFMs reporting IPV

When examining AFMs who reported IPV both SAB and AB had more females than males reporting IPV. SABs were mostly aged within the 25–34-year age and 35–49-year age ranges. The SEIFA pattern was similar to that observed for overall FV: SABs were more clustered around the top two quintiles, and ABs were spread more evenly across the second to top quintile. There was also a significant difference in the respondent’s country of birth, with a significantly higher proportion of SABs reporting a SAB respondent, whilst AB AFMs reported the highest proportion of Australian-born respondents.

A further examination of sex within relationships revealed that all SAB female AFMs reporting IPV had male respondents, whilst 90% of SAB male AFMs reporting IPV had female respondents. On the other hand, 98.1% of AB female AFMs reported a male IPV respondent, whilst AB male AFMs had 94.6% female IPV respondents.

#### Sample characteristics of AFMs reporting NPFV

Of the AFMs who reported NPFV, SABs were significantly less likely to be female than AB AFMs. There were no significant differences in the ages of AFMs reporting NPFV. There was a significant difference in the SEIFA levels reported by AFMs, again mirroring the pattern seen in AFMs reporting IPV and overall FV. There was also a significant difference in their respondent’s country of birth, with a significantly larger proportion of SABs reporting a SAB respondent, whilst AB AFMs reported the highest proportion of Australian-born respondents.

A further examination of sex within relationships revealed that SAB female AFMs reporting NPFV had a male respondent 75.8% of the time, and 92.9% of SAB male AFMs had a male respondent. On the other hand, AB female AFMs reporting NPFV had a male respondent 63% of the time, and 74.1% of AB male AFMs had a male respondent.

### Incidence rate

Based on all incidents reported to the police in the 6-month data collection period and using the estimated residential population of Australia in 2021 (based on census data), the 6-month incidence of the overall Victorian population was 499.23 per 100,000 Victorians. The SAB Victorian population’s incident rate for IPV was 79.45 cases per 100,000 SAB people, while the incidence of NPFV was 20.63 cases per 100,000 SAB people. On the other hand, the AB Victorian population had an IPV incidence of 224.09 cases per 100,000 AB people and 143.92 NPFV cases per 100,000 AB people.

### Revictimisation

Table 2 compared the likelihood of AFMs reporting revictimisation by any respondent and by the same or a different respondent in the 12 months following their reference incident. Overall, SAB individuals were significantly less likely to report revictimisation than AB individuals by any respondent, by their reference incident IPV respondent or by a different respondent to their reference incident. Differences in revictimisation reported by the same NPFV respondent in their reference incident were not significant between groups.

**Table 2.** Re-victimisation by AFM in the 12 months following reference incident.

	South Asian born		Australian born		χ^2^	*p*	*OR*	95% CI
	*N*	%	*N*	%				
Any FVR	80	21.1	271	36.1	25.36	<.001	0.48	[0.36, 0.64]
Same IPV dyad	68	22.3	244	40.0	28.36	<.001	0.46	[0.34, 0.62]
Same non-partner dyad	12	16.0	30	20.0	0.53	.468	0.91	[0.46, 1.82]
Different respondent	24	6.3	156	20.5	38.48	<.001	0.26	[0.17, 0.41]

Note: AFM = affected family member; FVR = Family Violence Report; IPV = intimate partner violence; *OR* = odds ratio; CI = confidence interval.

### Risk and vulnerability factors

#### Proportion of FVRs with a potential for a severe charge

There was not a significant difference between the two groups in the proportion of reference incidents classified as warranting a severe charge for overall FV, IPV and NPFV (Table 3).

**Table 3.** FVRs classified as severe based on the nature of associated charges.

Number of severe incidents	South Asian born		Australian born		χ^2^	*p*	*OR*	95% CI
	*N*	%	*N*	%				
Overall	54	14.2	99	13.0	0.31	.58	1.11	[0.77, 1.58]
Intimate partner	41	13.4	92	15.0	0.44	.51	1.10	[0.83, 1.44]
Non-partner	13	17.6	25	16.9	0.16	.90	1.05	[0.50, 2.19]

Note: Case-control matched sample. FVR = Family Violence Report; *OR* = odds ratio; CI = confidence interval.

#### Number of risk factors present

Table 4 indicates that there was no significant difference between groups in the number of risk factors recorded at the time of the reference FVR for overall FV incidents and NPFV specific reports. However, there was a significant difference in the number of risk factors reported for IPV incidents between the two groups, with SABs reporting a greater number of risk factors in their IPV incidents.

**Table 4.** Number of risk factors present in FVR.

Type of violence	South Asian born		Australian born		*U*	Z	*p*	*r*
	*Mdn*	*N*	*Mdn*	*N*				
Overall family violence	6	380	5.5	760	140,807.5	−0.69	.49	.02
Intimate partner violence	5	305	4	610	79,134	−3.71	.00	.12
Non-partner family violence	2	75	2	150	5,325.5	−0.67	.51	.04

Note: Case-control matched sample. FVR = Family Violence Report.

#### Frequency of recorded risk and vulnerability factors at the reference incident

Table 5 shows analyses testing for differences in the frequency of risk and vulnerability factors identified at the time of the reference incident, regardless of the nature of the FV relationship between AFM and respondent.

**Table 5.** Frequency of risk and vulnerability factors experienced for overall FV.

	South Asian born *N* = 380		Australian born *N* = 760		χ^2^	*p*	*OR*	95% CI
	*N*	%	*N*	%				
Affected family member factors								
Mental health problems	61	16.1	229	30.1	26.47	<.001*	0.44	[0.32, 0.61]
Isolated	95	25.0	111	14.6	18.49	<.001*	1.95	[1.43, 2.65]
Visa dependent	32	8.4	4	0.5	51.63	<.001*	17.38	[6.10, 49.52]
Respondent factors								
Mental health problems	69	18.2	332	43.7	72.39	<.001*	0.29	[0.21, 0.39]
Previously violent offence	56	14.7	383	50.4	136.04	<.001*	0.17	[0.12, 0.23]
Previous physical violence	183	48.2	358	47.1	0.11	.737	1.04	[0.82, 1.34]
Unemployed	59	15.5	252	33.2	39.70	<.001*	0.37	[0.27, 0.51]
Relationship factors								
Financial problems	84	22.1	204	26.8	3.10	.083	0.77	[0.58, 1.03]
Presence of children	183	48.2	322	42.4	3.44	.064	1.26	[0.99, 1.62]
Reside together	249	65.5	317	41.7	57.48	<.001*	2.66	[2.06, 3.43]
Recently separated	146	38.4	274	36.1	0.61	.435	1.11	[0.86, 1.43]
Abuse for >1 month	226	59.5	396	52.1	5.55	.019	1.35	[1.05, 1.73]
Violence escalated recently	174	45.8	345	45.4	0.02	.900	1.02	[0.79, 1.30]
Prior FV history	161	42.4	446	58.7	27.09	<.001*	0.52	[0.40, 0.66]
Risk factors for femicide								
Jealous/controlling actions	177	46.6	277	36.4	10.85	<.001*	1.52	[1.18, 1.95]
Ever threatened to harm/kill	73	19.2	172	22.6	1.76	.185	0.81	[0.60, 1.11]
Ever physically violent while pregnant	21	5.5	47	6.2	0.20	.658	0.89	[0.52, 1.51]
Ever strangled/suffocated	44	11.6	87	11.4	1.00	.948	1.01	[0.69, 1.49]
Ever sexually assaulted	18	4.7	31	4.1	0.27	.606	1.17	[0.65, 2.12]
Ever stalked/harassed	49	12.9	123	16.2	2.14	.144	0.77	[0.54, 1.10]

Note: FV = family violence; *OR* = odds ratio; CI = confidence interval.

*Bonferroni correction to *p* = .0025.

#### IPV risk and vulnerability factors

Table 6 explores the differences in risk and vulnerability factors related to the AFMs reporting IPV to the police.

**Table 6.** Frequency of risk and vulnerability factors for IPV.

	South Asian born *N* = 305		Australian born *N* = 610		χ^2^	*p*	*OR*	95% CI
	*N*	%	*N*	%				
Affected family member factors								
Isolated	88	28.9	99	16.2	19.93	<.001*	2.09	[1.51, 2.91]
Visa dependent	32	10.5	<10	<1.6%	46.36	<.001*	11.80	[4.88, 28.55]
Respondent factors								
Unemployed	42	13.8	173	28.4	24.08	<.001*	0.40	[0.28, 0.58]
Relationship factors								
Financial problems	72	23.6	153	25.1	0.24	.625	0.92	[0.67, 1.27]
Presence of children	160	52.5	255	41.8	9.32	.002*	1.54	[1.17, 2.03]
Reside together	211	69.2	241	39.5	71.62	<.001*	3.44	[2.57, 4.60]
Recently separated	144	44.7	265	43.4	1.17	.280	1.15	[0.88, 1.54]
Abuse for >1 month	196	64.3	313	51.3	13.82	<.001*	1.71	[1.27, 2.26]
Violence escalated recently	145	47.5	273	44.8	0.64	.425	1.12	[0.85, 1.47]
Risk factors for femicide								
Jealous/controlling actions	163	53.4	273	44.8	5.05	.013	1.42	[1.08, 1.87]
Ever threatened to harm/kill	57	18.7	155	25.4	5.16	.023	0.68	[0.48, 0.95]
Ever physically violent while pregnant	21	6.9	46	7.5	0.13	.720	0.91	[0.53, 1.55]
Ever strangled/suffocated	44	14.4	86	14.1	0.02	.893	1.03	[0.69, 1.52]
Ever sexually assaulted	18	5.9	30	4.9	0.4	.529	1.21	[0.67, 2.21]
Ever stalked/harassed	45	14.8	136	22.3	7.29	.007	0.60	[0.42, 0.87]

Note: IPV = intimate partner violence; *OR* = odds ratio; CI = confidence interval.

*Bonferroni correction to *p* = .003.

#### Non-partner FV risk and vulnerability factors

Table 7 explores the differences in risk and vulnerability factors related to the AFMs reporting NPFV to the police.

**Table 7.** Frequency of risk and vulnerability factors for NPFV.

	South Asian born *N* = 75		Australian born *N* = 150		χ^2^	*p*	*OR*	95% CI
	*N*	%	*N*	%				
Affected family member factors								
Isolated	<10	<13.3%	13	8.7	0.24	.627	1.26	[0.50, 3.18]
Relationship factors								
Financial problems	12	16	34	22.7	1.37	.242	0.65	[0.31, 1.34]
Reside together	38	50.7	53	35.3	4.88	.027	1.88	[1.07, 3.30]
Abuse for >1 month	30	40	70	46.7	1.18	.343	0.76	[0.43, 1.34]
Violence escalated recently	29	38.7	59	39.3	0.01	.923	0.97	[0.55, 1.72]
Risk factors for femicide								
Jealous/controlling actions	14	18.7	15	10	3.34	.067	2.07	[0.94, 4.55]
Ever threatened to harm/kill	16	21.3	23	15.3	1.26	.262	1.50	[0.74, 3.04]

Note: NPFV = non-partner family violence; *OR* = odds ratio; CI = confidence interval.

*Bonferroni correction to *p* = .007.

## Discussion

This study aimed to compare family violence reports (FVRs) made to Australian police by South Asian migrants with reports made during the same period by a population of people born in Australia.

### Who seeks help?

The first aim of this study was to describe the demographic characteristics of those who sought help from the police. There were no differences between the two groups in the sex of AFMs and respondents, though as hypothesised, more females were recorded as AFMs in both groups. This is consistent with many studies demonstrating that women are more likely to be victims of FV than men (Australian Institute of Health & Welfare, AIHW, 2018; Garcia-Moreno et al., 2006). There were significant age and SEIFA differences between the SAB and AB groups, with the SAB AFMs being slightly younger. This mirrors age data of the two populations from the 2021 Census, where AB Australians (*M* = 37) were slightly older than SAB individuals (*M* = 35) (ABS, 2022b). There were also SEIFA differences between the two groups. This likely reflects underlying socioeconomic differences in the Australian population, where SAB individuals have a higher reported weekly income than AB individuals (ABS, 2022b).

The hypothesis that females would report IPV and NPFV in both groups was partially supported. Both groups showed females reporting more IPV. However, a significant difference emerged in the proportion of female AFMs reporting NPFV, with more male SABs report NPFV. In the few studies that examine NPFV amongst South Asians, often the focus has been on female victimisation by female perpetrators, mainly daughter-in-law abuse by a mother-in-law (Gangoli & Rew, 2011; Kandiyoti, 1988; Martin et al., 2013; Raj et al., 2006, 2011). However, Rai and Choi (2022) identified this gap in the literature and sampled both male and female South Asians across the United States. Upon finding that South Asian men and women reported similar rates of FV by in-laws (19% and 21%, respectively), the authors acknowledged that further research into South Asian male experiences of FV is essential (Rai, 2021; Rai & Choi, 2022; Ravi et al., 2023). The current study found that 9 out of 10 SAB male victims of NPFV were reporting victimisation by a male family member. Similarly, nearly three quarters of SAB women who reported NPFV victimisation were reporting victimisation by a male family member. Further examination of the NPFV found that male parents made up a third of all SAB NPFV’s reported respondents. These results indicate that further research is needed on NPFV. Specifically, this study highlighted gaps in understanding South Asian male experiences of NPFV as well as South Asian male perpetration of NPFV, particularly by fathers towards their sons.

### Six-month incidence rate of reported family violence and revictimisation

The hypothesis that SAB individuals would have a lower incidence rate of police-reported FV than AB individuals was supported. This is consistent with anecdotal observations by FV service providers and stakeholders in the jurisdiction where the research was undertaken, who have noted that Indian women use FV services less than would be expected relative to the size of their population (Colucci et al., 2021). This could be due to less FV amongst South Asian migrants, or it may reflect difficulties accessing services. The broader literature provides more support for the latter explanation, with multiple reasons for under-reporting among South Asian migrants being documented (Dasgupta, 2000; Mahapatra, 2012; Mahapatra & DiNitto, 2013; Mahapatra & Rai, 2019; Rai & Choi, 2022; Raj & Silverman, 2007; K. Tonsing & Tonsing, 2019). This finding is the first quantitative evidence that suggests under-reporting within a South Asian Australian population, although Easteal (1996) did suggest that migrants in Australia are likely to be under-represented in police data as they are less likely to see police as an option. However, to confirm the under-reporting hypothesis, additional epidemiological research would be needed that adequately samples a representative group of South Asian Australians (De Silva et al., 2023a, 2023b).

The hypothesis that SAB individuals under-reporting to the police would lead to lower rates of revictimisation was also supported. The reporting of revictimisation may have been influenced by prior experiences of police contact. On one hand, police intervention during the reference incident may have been successful at reducing or eliminating the occurrence of FV for South Asian Australians, rendering future police contact as redundant. On the other hand, lower rates of re-reporting may be because of general under-reporting or that the police intervention at the reference incident was not considered helpful and so police were not contacted further. Further investigation around perceptions of police contact amongst SAB Australians would help clarify whether under-reporting is influenced by negative police experiences or fear of discrimination.

### Comparative severity of FV reported by SAB and AB populations

Based on the conclusions of prior qualitative research, it was hypothesised that when South Asian people did report FV to police, they would report experiences of greater severity. This reflects a hypothesis that people of South Asian backgrounds turn to formal supports when they perceive few other options. Contrary to this hypothesis, there was no evidence that the reference incident involved more severe behaviour, based on the types of charges that police identified as appropriate at the time of the incident. In both the SAB and AB samples, approximately one in seven cases had evidence of ‘severe’ FV based on the presence of charges for a significant violence or psychological abuse (stalking or threats). This novel finding could suggest that if South Asians do delay seeking police support, as previously international research suggests, when they do seek help, the level of severity of the incident is not different.

There are, however, potential alternative explanations for this unexpected finding. As noted above, concerns around discriminatory practices and unwanted outcomes may be barriers to help-seeking (Belur, 2008; Ghafournia & Easteal, 2021; Hague et al., 2010; Izzidien, 2008; Merchant, 2000; Rai & Choi, 2022; Raj & Silverman, 2002, 2007; Sultana et al., 2023). Migrant women in Australia have reported a reluctance and/or fear of informing law enforcement due to uncertainty around the scope of domestic violence legislations, particularly as migrants may have had negative experiences in their countries of origin (Block et al., 2022; Easteal, 1996; Rajkhowa et al., 2022). It is possible that such concerns mean that SAB AFMs are less willing to disclose severe experiences to a police officer. Language barriers may have a similar effect, preventing AFMs from communicating clearly about what has happened (Belur, 2008; Femi-Ajao et al., 2020; Hulley et al., 2023). This has previously been observed in an Australian study of immigrant women in Australia (Easteal, 1996). It is also possible that police charging practices might differ for different cultural or racial groups, leading to lower charge levels among SABs. Research in the United States indicates that reports by minority victims are less likely to result in an arrest (McCormack & Hirschel, 2021; Winstead & Stevenson, 2022). There is no equivalent Australian policing data, meaning it is unclear whether race, ethnicity or cultural background are likely to have influenced charging decisions (Dowling et al., 2018).

The second measure of severity was the number of risk and vulnerability factors present at the reference incident. IPV cases involving SAB AFMs had more risk and vulnerability factors present at the time of the reference incident, suggesting that they may be a higher risk cohort. Given that higher levels of revictimisation reporting would be expected in a higher risk cohort, this finding provides some support for the under-reporting hypothesis previously discussed. It may be that despite being at higher risk of further FV, all of the barriers to reporting mean that further victimisation is similarly under-reported. Alternatively, it is possible that the SAB AFMs experienced more repercussions for reporting to police once, and so were less likely to do so again. It is also possible that they found that the police and FV sector response was not useful, and so did not turn to formal supports when experiencing further FV (Ghafournia & Easteal, 2021). There were a similar number of risk and vulnerability factors present at the reference incident for AB and SAB groups when FV was examined overall, and when examining NPFV.

### Frequency of risk factors for overall FV, NPFV and IPV

This paper also attempted to examine whether SAB were more likely than AB participants to have specific risk factors that have been associated in prior research with FV generally and specifically with IPV or NPFV.

#### Overall FV

Examining risk factors associated with police-reported FV generally, more SAB AFMs reported the victim vulnerability factors of isolation and visa dependency when reporting overall FV incidents. As discussed above, visa dependency is not only a barrier to seeking help, but can also be used as a tool for abuse (Midlarsky et al., 2006; Segrave, 2017). Similarly, Abraham (2000) argues that ‘isolation is one of the most painful manifestations of marital abuse perpetrated against [South Asian immigrant women]’ (p. 222), which can trap women in the abusive relationship for longer. Other research has also indicated that isolation can be used as a technique of abuse by both intimate partners and non-partner family members (Abraham, 2005; Ghafournia, 2011; Sabri & Young, 2022; Singh, 2019; Singh & Sidhu, 2020).

SAB AFMs were significantly less likely to report the presence of mental health problems for themselves or their respondent. This could reflect a genuine difference between groups, or alternatively might reflect unwillingness to report mental health issues due to stigma (Ekanayake et al., 2012; Islam et al., 2014; Kapadia et al., 2017; Prajapati & Liebling, 2022; Sharma et al., 2020). It is also possible that different cultural concepts of mental health could lead to different reporting patterns among the South Asian cohort. In Karasz et al.’s (2019) literature review, South Asians in the United Kingdom, Canada and South Asia perceive mental illness through more social frameworks rather than a bio-medical framework often used by Western medical professionals. A mismatch in conceptualisation may have led to fewer reports of mental health problems to a police officer.

Two risk factors that had a significantly lower frequency of reports by SAB AFMs were respondents who had a prior violent offence and those who had a history of FV. These results may well reflect under-reporting of FV generally in this group, as previously discussed. Alternatively, as migrants have not lived in Australia their whole life, it is likely that they have had less opportunity to have reports recorded by the police than AB individuals.

Despite SEIFA being controlled for by the matched sample, SAB individuals were significantly less likely to have an FV respondent that was unemployed. This may again be a reflection of underlying socioeconomic differences between the two groups. SEIFA levels are a geographical indication of socioeconomic status; SABs may still demonstrate a higher socioeconomic status than ABs generally. For example, SABs were found to have a higher reported weekly income than AB individuals in the 2021 Census (ABS, 2022b).

Within relationship factors, residing together was more frequent for SABs. This was an expected finding due to South Asian cultural values of collectivism and familism that put a high importance around family and is further explored below.

Having a jealous and controlling respondent was the only behavioural risk factor that differentiated between the two groups, with SAB AFMs having a higher frequency for overall FV. This factor was included due to its association with severe outcomes in a non-SAB sample (Spencer & Stith, 2020). The SAB group reported this more often the AB group. The literature does demonstrate that jealousy and control are related to severe physical IPV and injuries, a finding identified in a nationally representative sample of women in India (Sabri et al., 2014), and contributed to severe IPV within a sample of Asian migrants and refugees in the United States (including Indian and Pakistani individuals; Sabri et al., 2018). Controlling behaviours could be discussed as related to gender roles and expectations. As South Asian cultures are considered to be patriarchal, male dominance over females is often accepted and normalised (Ayyub, 2000; Simister & Mehta, 2010). Typically, however, the normalisation of these roles means that South Asian women do not classify themselves as being ‘controlled’, thus it is unlikely for those who hold such beliefs to report that their partner is controlling (underestimating the overall number). More research should be done to understand whether there is a ‘tipping point’ when SABs realise the level of control they are subjected to, or whether those who report controlling partners hold less patriarchal views – that is, they are more acculturated. It is important to note, however, that studies are often difficult to compare due to the lack of standardised measures across these studies. For example, in the Sabri et al. (2014) study, jealousy and controlling behaviours were found to be significant as two separate risk factors, unlike the combined nature of the factor within this study. Further research should consider taking a more standardised approach to measure these variables and examine them separately.

#### IPV

This study hypothesised that SAB individuals reporting IPV would demonstrate that they were more likely to be isolated, be visa dependent and be abused for longer than a month. They were also hypothesised to be more likely to have children present, reside together or report experiencing escalating violence. The results mostly supported these hypotheses.

Similar to the results found for overall FV, SAB individuals reporting IPV demonstrated a higher frequency of isolation, visa dependency and living together. The rationale for isolation and visa dependency are as discussed above. These results may be simply a reflection of South Asian culture and intimate relationships. Alexander and colleagues (2006) state that a ‘hint of a pre-marital relationship [by parents] can hasten marriage for young women’ (p.144), as dating is not a common practice in South Asia and is often discouraged (Alexander et al., 2006; Couture-Carron, 2017). Given the practice of the bride moving out of her family home and into the groom’s family home after marriage (Ahmad-Stout et al., 2021; Ali et al., 2021; Gangoli & Rew, 2011), it is likely that a larger frequency of SAB individuals are married and live together. A further examination of the breakdown of IPV relationships in this dataset found that that two thirds of SABs reporting IPV reported a respondent they were married to.

In general, cohabitating couples are known to be more likely at risk regardless of relationship status – for example, married or dating (Capaldi et al., 2012). Additionally there is huge stigma against divorce within South Asian communities, which makes such relationships less likely to occur and thus reported (J. Tonsing & Barn, 2017).

SABs reported a higher frequency of children present. The chances of having children present are likely to increase if the relationship consists of two people living together, which may be related to the significant difference seen in the reported presence of children in IPV incidents. Alternatively, many South Asian women have qualitatively reported children as one of the key reasons they sought police help as they felt their children were in danger (Ahmad et al., 2009; Bhandari, 2018).

SABs reported a higher frequency of experiencing IPV for longer than a month. The literature supports these findings by indicating that violence is often denied and/or endured for a while before seeking help, especially when seeking help from the police. For example, Mahapatra and Rai’s (2019) sample of nine South Asian women in the United States remained in an abusive situation for a minimum of 2 years before engaging in any help-seeking (including informal help-seeking). Moreover, Ahmad et al.’s (2009) qualitative interviews of 22 South Asian immigrant women in Canada identified many reasons for the delay in seeking professional help for IPV, one of which was limited knowledge of resources. Issues in Australia’s service responses can be seen when combining these findings with the significantly higher number of risk factors present, and the lower likelihood of reporting revictimisation. These results indicate that SAB individuals are in unsafe situations for longer and experience a higher level of severity yet may find the police inaccessible. Thus, it is imperative to continue to examine specific barriers that South Asian Australians may face and identify ways to minimise these barriers to facilitate access to support.

There were no significant differences between the two groups when reporting that their IPV experience had escalated recently. This is an unexpected finding given the literature stating that South Asians mostly only report to the police when the violence has escalated (Bhandari, 2018; Ghafournia & Easteal, 2021). It is unclear whether these results were a consequence of how information was gathered by the police and reported by the AFM. Further research should attempt to identify whether a similar turning point of escalated violence is relevant to South Asian Australians.

This study also hypothesised that SABs would be more likely to report risk factors associated with IPV femicide (Spencer & Stith, 2020). There was no significant difference between the two groups, indicating similar frequencies experienced by SABs and ABs. These risk factors are used to help inform risk assessments for police (Petersson & Thunberg, 2022; Spencer & Stith, 2020) and as stated have been linked to IPV feminicide. It is worth examining whether these risk factors are specifically relevant in predicting severe violence outcomes, including feminicide, for South Asian Australians. This is particularly important, as there are additional concerns amongst South Asian communities over extreme manifestations of patriarchal power via dowry abuse and honour-based killing that are known to exist (Gill, 2014, 2017; Lima et al., 2020; Sabri et al., 2014)

#### NPFV

This study hypothesised that SAB individuals would report a significantly higher frequency of isolation, being financially strained, experiencing abuse for longer than a month, residing with their respondent, reporting jealous and controlling behaviours and experiencing escalating violence. None of these hypotheses were supported, with SAB AFMs demonstrating a similar frequency of these risk factors to AB AFMs. This is an unexpected finding particularly due to two populations having a significant sex difference in their AFM reporting FV. The identification of more SAB males as AFMs and fathers as respondents identifies a gap in the literature in understanding NPFV amongst South Asians. Further research is needed to fill this gap as well as examine what risk factors may be associated with severe outcomes within NPFV incidents.

### Limitations

There are four main limitations of this study. The use of administrative data recorded by police is inherently limited, and in this case the high levels of missing data related to country of birth was particularly problematic. It is possible that secondary use of a dataset means that the quality of the data is less reliable as police may not have been able to ascertain information reliably or were not able to ask all questions due to operational demands.

Secondly, due to the known barriers surrounding police help-seeking, it was of particular interest to identify in what circumstances police help was sought. Yet the validity of this exploration is upheld only by the assumption that the police were contacted by the AFMs themselves, rather than by witnesses such as neighbours. A valuable addition to police reporting, therefore, would be to identify who made initial contact and for future research to consider examining the differences between the reports made by the AFMs themselves or by others. Such insight may provide clarity over which incidents were specifically deemed to require police attention by the AFM.

Third, the dataset was collected by Victoria Police, a police force within one state of Australia. Compared to other states and territories in Australia, Victoria has invested a significant proportion of resources throughout the years to raise awareness of FV and implement prevention and intervention strategies. As stated, the Victorian Royal Commission into Family Violence (VRCFV) helped raise awareness and increased reporting; thus, Victorians may have a greater awareness of what FV is and whom to contact – for example, the police – than other states. As a result, the reporting rates may be higher than in other states. However, the incidents captured in this dataset are more likely to be representative of the actual number of incidents experienced than any other police dataset, and thus the results may be the most accurate depiction of police-reported FV.

Finally, the term South Asian refers to a very heterogenous group of individuals, who come from various cultural, religious, ethnic and social backgrounds, with numerous languages spoken amongst them. Lumping individuals into a ‘South Asian’ group is problematic as it oversimplifies their diversity and marginalises many who do not fit the generalisations made. Caution is therefore needed when examining FV trends amongst this group. However, there are benefits to using this collective term. At present there is still a trend of South Asians being included in a broader categorisation of ‘Asian’. Therefore the distinction of South Asian begins the disaggregation of larger sub-populations. Currently, South Asians are a recognised group for research purposes within Western countries where they are often the minority group, thus there is strength in the numbers as they share more similarities than other sub-populations (Barrett et al., 2020; Bhandari, 2018; Finfgeld-Connett & Johnson, 2013; Gill & Harrison, 2016; Rai & Choi, 2022).

### Conclusion

This study is the first quantitative examination of FV amongst South Asian Australians, as well as the first known examination of police-reported FV by South Asian migrants. Due to the dearth of literature, particularly using a comparative design, this study aimed to identify whether there were differences between police-reported FV incidents by South Asian migrants and those by AB individuals. First, the study sought to describe the demographic factors of those who sought help, and second to identify differences in the rate of reporting to the police. The third study aim was to determine whether differences exist in the level of severity reported by the two groups. The final aim of the study was to examine the prevalence of various risk and vulnerability factors recorded by the police in the two populations.

Findings confirmed group differences in the demographic, incident rate and revictimisation rate of FV though there was limited support that the severity of reported violence was greater in the South Asian population. SAB individuals reporting IPV seemed to be at higher risk than AB individuals due to a greater number of risk factors present at the time of the incident. However, other measures of severity seemed to indicate similarities with ABs. There were some risk and vulnerability factors recorded by the police that were more common amongst SAB individuals, and others that were more common amongst AB individuals. Several recommendations on how to extend this area of research beyond this current study were identified. Further research in this area will help to build an evidence base that can guide effective policy and practice for South Asians, both in Australia and globally.
